# 贝林妥欧单抗联合达沙替尼逆转Ph阳性急性淋巴细胞白血病患者抗CD19 CAR-T细胞治疗后融合基因转阳1例

**DOI:** 10.3760/cma.j.issn.0253-2727.2022.08.015

**Published:** 2022-08

**Authors:** 瑞华 米, 琳 王, 琳 陈, 佳 刘, 旭东 魏

**Affiliations:** 郑州大学附属肿瘤医院/河南省肿瘤医院，郑州 450008 The Affiliated Cancer Hospital of Zhengzhou University/Henan Cancer Hospital, Zhengzhou 450008, China

患者，女，53岁，以“确诊急性淋巴细胞白血病2个月余，化疗2周期后”为主诉于2016年7月6日第1次来我院就诊。2016年4月14日患者因“发热10余天，加重伴头晕、黑便2 d”就诊某医院，入院后的血常规示：WBC 38.6×10^9^/L，HGB 63 g/L，PLT 13×10^9^/L；骨髓象：增生明显活跃，原始幼稚细胞占83％；流式细胞术检测免疫表型：72.91％细胞（占有核细胞）表达CD34、TdT、CD10、CD20，部分表达CD33、HLA-DR、CD13、CD19、CD22，不表达CD117、CD15、CD11b、CD16、CD14、CD64、CD11c、CD4、CD8、CD56、CD2、CD5、CD7、cCD3、MPO；染色体核型：45～46，XX，t（9;22）（q34;q11），+der（9）t（9;22），+16，−22，der（22）t（9;22）[cp5]；FISH：BCR-ABL融合基因阳性细胞比例为75％，未见到分子生物学的检查结果。诊断为急性淋巴细胞白血病伴Ph^+^，高危组。2016年4月19日给予VTCP（具体剂量不详）方案诱导缓解治疗，1个周期后疗效评价未缓解（NR）。2016年6月2日骨髓象：增生活跃，原始幼稚淋巴细胞占41％，于2016年6月3日再次给予VDCP（具体剂量不详）联合甲磺酸伊马替尼400 mg/d治疗。

2016年7月6日我院检查结果示：骨髓象：增生明显活跃，幼稚淋巴细胞占0.2％；BCR-ABL210/ABL^IS^：2.3％；染色体核型：46，XX[20]；流式细胞术检测微小残留病（MRD）示未见幼稚B淋巴细胞。疗效评价为完全缓解（CR）。2016年7月至2020年11月期间患者接受多周期化疗及联合酪氨酸激酶抑制剂（TKI）治疗，BCR-ABL融合基因持续处于阴性状态。

2020年11月3日再次入院，血常规：WBC 3.12×10^9^/L，HGB 106 g/L，PLT 157×10^9^/L；骨髓象：增生尚活跃，未见原始幼稚淋巴细胞；流式细胞术MRD示阴性；BCR-ABL210/ABL^IS^：0.1％，ABL激酶区突变检测未发现突变位点，提示分子学复发，患者及家属拒绝异基因造血干细胞移植（allo-HSCT），后于2020年11月10日给予氟马替尼（商品名昕福）600 mg/d口服治疗。

2020年11月25日骨髓象：增生尚活跃，未见原始幼稚淋巴细胞；流式细胞术MRD：异常B淋巴细胞占0.06％；BCR-ABL210/ABL^IS^: 1.47％。追问患者服药情况，自行在家服用氟马替尼200～400 mg/d，后将药物加量至600 mg/d。2020年12月7日骨髓象示增生活跃，未见原始幼稚淋巴细胞，流式细胞术MRD示阴性，BCR-ABL210/ABL^IS^：0.037％，后院外一直服用氟马替尼600 mg/d治疗。

2021年4月20日血常规：WBC 2.2×10^9^/L，HGB 89 g/L，PLT 126×10^9^/L；骨髓象：增生减低，幼稚淋巴细胞6％；流式细胞术MRD：CD19^+^异常幼稚B淋巴细胞占17.26％；BCR-ABL210/ABL^IS^：42.35％。后采集患者外周血的淋巴细胞，拟给予抗CD19 CAR-T细胞治疗。2021年4月26日骨髓象：增生尚活跃，幼稚淋巴细胞36.8％。按治疗计划于2021年5月30日回输CAR-T细胞0.5×10^8^，细胞回输后仅发生Ⅰ级发热，余未见明显的不良反应。细胞回输后第15天时复查骨髓融合基因示阴性，后融合基因持续阴性维持半年余。

2021年12月2日骨髓象示增生减低，未见原始幼稚淋巴细胞，流式细胞术MRD示阴性，BCR-ABL210/ABL^IS^：0.055％，ABL激酶区突变检测示阴性，提示分子学复发，即退出临床研究，给予口服达沙替尼。

2021年12月22日血常规：WBC 3.23×10^9^/L，HGB 116 g/L，PLT 109×10^9^/L；骨髓象：增生尚活跃，未见原始幼稚淋巴细胞；流式细胞术MRD：共获取有核细胞约500 000个，其中CD19^+^CD10^++^ CD20^−^CD34^part+^CD38^dim+^CD81^dim+^CD33/CD13^dim+^CD45^dim+^异常幼稚B淋巴细胞占0.05％；BCR-ABL210/ABL^IS^：0.33％，ABL激酶区突变检测示阴性，融合基因明显升高。于2021年12月27日给予贝林妥欧单抗联合达沙替尼治疗，具体为：贝林妥欧单抗（商口名：倍利妥）9 µg第1～7天，28 µg第8～22天，达沙替尼（商品名：施达赛）50 mg/d口服逐渐爬坡至50 mg口服每日2次患者耐受剂量。贝林妥欧单抗治疗过程中，未见细胞因子释放综合征等不良反应的发生，耐受性良好。贝林妥欧单抗应用第15天时骨髓象：增生尚活跃，未见原始幼稚淋巴细胞；流式细胞术MRD示阴性，BCR-ABL210示低于最低检出下限，院外达沙替尼单药治疗。

2022年2月17日血常规示WBC 4.21×10^9^/L，HGB 93 g/L，PLT 44×10^9^/L；骨髓象示增生减低，未见原始幼稚淋巴细胞；流式细胞术MRD示阴性；BCR-ABL210低于最低检出下限。再次给予贝林妥欧单抗28 µg/d持续静脉输注×14 d治疗。目前该患者的融合基因再次维持阴性3个月余，长期疗效持续随访中。

